# One-Pot Approach to Prepare Organo-silica Hybrid Capillary Monolithic Column with Intact Mesoporous Silica Nanoparticle as Building Block

**DOI:** 10.1038/srep34718

**Published:** 2016-10-04

**Authors:** Shengju Liu, Jiaxi Peng, Zheyi Liu, Zhongshan Liu, Hongyan Zhang, Ren’an Wu

**Affiliations:** 1 CAS Key Laboratory of Separation Sciences for Analytical Chemistry, National Chromatographic R&A Center, Dalian Institute of Chemical Physics, Chinese Academy of Sciences (CAS), Dalian 116023, China; 2 University of Chinese Academy of Sciences, Beijing 100049, China

## Abstract

A facile “one-pot” approach to prepare organo-silica hybrid capillary monolithic column with intact mesoporous silica nanoparticle (IMSN) as crosslinker and building block was described. An IMSN crosslinked octadecyl-silica hybrid capillary monolithic column (IMSN-C18 monolithic column) was successfully prepared, and the effects of fabrication conditions (e.g. concentration of intact mesoporous silica nanoparticle, polycondensation temperature, content of vinyltrimethoxysilane and stearyl methacrylate) on the structures of the IMSN-C18 monolithic column were studied in detail. The IMSN-C18 hybrid monolithic column possessed uniform morphology, good mechanical and pH stability (pH 1.1–11), which was applied to the separations of alkyl benzenes, polycyclic aromatic hydrocarbons (PAHs), as well as proteins. The minimum plate height of 10.5 *μ*m (corresponding to 95000 N m^−1^) for butylbenzene and high reproducibility were achieved. The analysis of tryptic digest of bovine serum albumin (BSA) was carried out on the IMSN-C18 monolithic column by cLC coupled mass spectrometry (cLC-MS/MS), with the protein sequence coverage of 87.5% for BSA, demonstrating its potential application in proteomics.

Monolithic columns have been applied in liquid chromatography and suggested as the great alternatives to the particulate-packed and open-tubular columns in separation sciences since their easy preparations, high performances and the versatile functionalities^1^,^2^,^3^,^4^,^5^,^6^,^7^,^8^,^9^,^10^. Based on chemical nature of precursors applied, monolithic columns can be generally classified into organic polymer-based monoliths, inorganic silica-based monolithic columns and organic-inorganic hybrid monolithic columns^2^,^7^,^8^. Among them, the organic‒inorganic hybrid monoliths not only have the advantages of polymer-based monoliths and silica-based monolithic columns, such as easy preparation, high pH tolerance and good mechanical stability^10^,^11^,^12^, but also avoid the drawbacks of solvent swelling and tedious functionalization^9^,^12^,^13^,^14^. Therefore, the organic‒inorganic hybrid monoliths have received increasing attention in recent years.

The incorporation of the organic precursor N-octadecyldimethyl[3-(trimethoxysilyl)propyl]ammonium chloride (C18-TMS) into the reaction solution along with tetramethoxysilane (TMOS) was achieved by Hayes and Malik to prepare an organic-inorganic hybrid monolithic column for CEC separation^15^. Through this method, organic-silica hybrid monoliths with various functional moieties, such as aminopropyl, octyl, phenyl, vinyl, allyl and propyl etc^16^,^17^,^18^,^19^,^20^,^21^,^22^,^23^. have been fabricated and applied in chromatographic separations as well as solid phase extractions (SPEs)^24^. However, the use of traditional sol-gel method to prepare the silica-based hybrid monolithic column was hindered due to the limited types and difficulties in the synthesis of organo-trialkoxysilanes. Therefore, a “one-pot” approach, in which vinyl-organic monomers are concurrently introduced into capillary with pre-hydrolyzed tetramethyl orthosilicate (TMOS) and vinyltrimethoxysilane (VTMS) to go through polycondensation and successive polymerization, has been developed to circumvent the above limitations^25^. Based on the advanced “one-pot” strategy, a variety of organic-silica hybrid monolithic columns with phenyl^26^, butyl^27^, ion liquids^28^,^29^, zwitterion^30^, phenylboronic acid^31^ and β-cyclodextrin^32^ functional groups have been prepared for reverse-phase, hydrophilic-interaction and/or chiral chromatography to separate small molecules and/or biomacromolecules. Other than the free radical copolymerization, organo-functional groups could also be incorporated into silica monolithic matrix via epoxy ring-opening reaction^33^ and thiol-ene click reaction^34^,^35^,^36^.

Besides the above-mentioned advances on governing the functionalities of organic-silica monoliths with specific organic monomers, the approach of using a nanometer-sized particle of polyhedral oligomeric silsesquioxanes (POSS)^37^ as the building block has been progressed to construct the nanophase-containing organic-inorganic hybrid monolith through copolymerization^38^,^39^,^40^,^41^. Benefited from the nanophase technology, the nano-hybrid silica-based monolithic column demonstrated well 3D structure, enhanced mechanical and/or thermal stability. In this work, the intact mesoporous silica nanoparticle (IMSN, the as-synthesized mesoporous silica nanoparticle without removal of templates) was utilized as building block and crosslinker to prepare the IMSN crosslinked organo-silica hybrid monolithic column through the “one-pot” approach. An IMSN-C18 hybrid capillary monolithic column has been successfully fabricated by using stearyl methacrylate (C18 monomer) as the organic monomer. The prepared IMSN-C18 organic-inorganic hybrid capillary monolithic column was systematically investigated, which demonstrated high column performance, uniform structure, good mechanical and pH stability. The nanoparticle-based “one-pot” method with the preformed IMSN as building block and crosslinker would represent a new and convenient way to prepare the silica-based hybrid monolithic column for further wide applications.

## Results

### Optimization of the IMSN-C18 monolith

**Figure 1:**
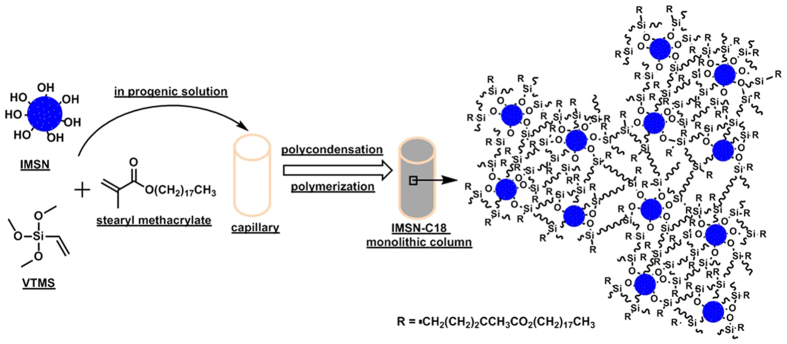
Scheme for preparation of IMSN-C18 organic-inorganic hybrid monolithic column.

Based on the “one-pot” approach^25^, the IMSN-C18 hybrid monolithic columns were prepared by using the IMSN as crosslinker and building block. The schematic process of the IMSN-C18 organic-inorganic hybrid monoliths is illustrated in Fig. 1. The procedure involves two major reactions: the polycondensation of IMSN with hydrolyzed VTMS to form the monolithic silica skeleton, and the in-situ free-radical copolymerization of the incorporated vinyl moieties on the as-synthesized silica skeleton with the C18 monomer. As the morphology of monolithic column can be tuned by changing conditions such as the composition of the polymerization mixture and the reaction temperature^25^,^26^,^27^, several synthesis parameters affecting the formation of IMSN-C18 monoliths were evaluated. The detailed fabrication conditions and the results are illustrated in Table 1.

**Figure 2:**
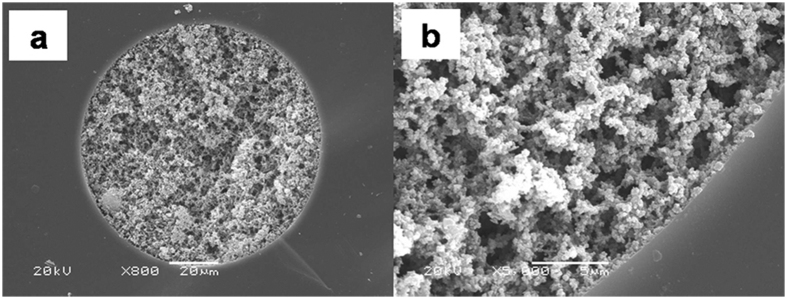
SEM images of IMSN-C18 organic-silica hybrid monolithic column C. Magnification. (**a**) × 800, (**b**) × 5000.

As the amount of nanoparticle would affect skeleton formation and permeability of monolithic column^42^,^43^, the concentration of IMSN ranging from 15 to 35 mg mL^−1^ was thus carefully investigated for the preparation of the IMSN-C18 hybrid monolithic columns. Optical microscope images of the monolithic columns fabricated with 15, 25 and 35 mg mL^−1^ of IMSN, along with other synthesis parameters, are displayed in Table 1. As seen from the optical microscope images, monolithic columns A, B and C were all homogeneous within the confinement of capillaries. Whereas, the morphologies of IMSN-C18 hybrid monolithic columns A and B were not as homogenous as that of column C based on the scanning electron microscope images (SEM) (Supplementary Fig. S1a,b, and Fig. 2). Moreover, the permeabilities of columns A, B and C decreased from 4.54 to 1.31 × 10^−14^ m^2^ as the concentration of the IMSN was increased from 15 to 35 mg mL^−1^. Thus, it is important to intensively tune the concentration of IMSN to prepare the IMSN crosslinked C18 hybrid monoliths.

Since the polycondensation of sol-gel reaction is temperature sensitive^25^, the condensation temperature in the synthesis of IMSN-C18 hybrid monolithic column was examined. As shown from microscopy images (Table 1), there was little difference among the morphologies of columns C, D and E fabricated at 40 °C, 35 °C and 45 °C, respectively. The comparison of SEM images shows that hybrid monolithic matrixes of column D and E were not as uniform as that of column C (Supplementary Fig. S1c,d, and Fig. 2). In addition, the column efficiency of the column C (95000 N m^−1^) was higher than those of columns D and E (69000 and 64000 N m^−1^) by cLC evaluation. Therefore, the adjustment of the temperature is vital to prepare the IMSN crosslinked hybrid monolithic column with good pore structure and high column efficiency. In this work, 40 °C was chosen to prepare other IMSN-C18 hybrid monoliths with different synthesis conditions.

Porogen has a significant effect on the morphology and porosity of the resulting hybrid monolith, so the amount of poly(ethylene glycol) (PEG, Mw = 10000) was optimized by comparing columns C, F and G (Table 1). As seen from the optical microscope images, the lower amount of PEG (2.5 mg, column F) would result in slack monolith with opaque aggregation inside the capillary, and the higher amount of PEG (7.5 mg, column G) could result in slight detachment of monolithic matrix from the inner capillary wall. However, the column C using 5 mg of PEG in the pre-polycondensation mixture exhibited the most homogeneous morphology. For investigating the influence of VTMS content on the morphologies of the IMSN-C18 hybrid monolithic column, 54, 104 and 154 *μ*L of VTMS were added to the reaction mixture, respectively. The morphologies of the resultant monolithic columns (column H, C and I) are displayed in Table 1. The optical microscopy images show that monolithic matrix of column H was detached from the inner wall of the capillary, which could be ascribed to the incomplete condensation of IMSN with 54 *μ*L of VTMS. While, the monolithic matrix was not homogeneous in capillary as the volume of VTMS was increased to 154 *μ*L (column I). The reason was that the excessive VTMS led to inhomogeneous reaction of IMSN and VTMS, and further resulted in the detachment of the monolithic matrix from the inner wall of capillary. However, the column prepared with 104 *μ*L of VTMS (column C) exhibited homogeneous morphology and rational back pressure.

For fabricating the IMSN crosslinked C18 organo-silica hybrid monolithic column, the C18 monomer was simultaneously mixed with the IMSN and VTMS, and the amount of C18 monomer in the reaction mixture was investigated. The microscope images (Table 1) show that the morphologies of columns prepared with different content of C18 monomer were homogeneous. However, the SEM morphology of column C (Fig. 2) synthesized with 30 *μ*L of C18 monomer was more homogenous than those of columns J and K with 15 and 45 *μ*L of C18 monomer (Supplementary Fig. S1e,f), respectively. As a result, the optimal preparation conditions for column C were employed in the following separations.

### Pore structure characterization of the IMSN-C18 monolith

**Figure 3:**
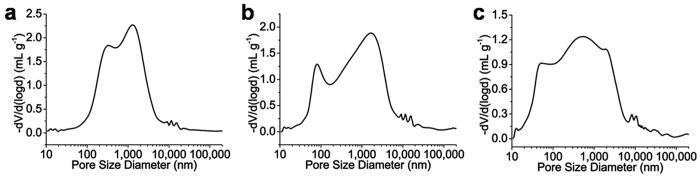
Pore size distribution of IMSN-C18 monoliths A (**a**), B (**b**), C (**c**) by the mercury intrusion method.

N_2_ adsorption/desorption and mercury intrusion porosimetry experiments were taken to characterize the pore structure of the IMSN-C18 hybrid monolith. The results are illustrated in Fig. 3, Supplementary Table S1 and Supplementary Table S2. The BET surface area (showed in Supplementary Table S1) of the IMSN-C18 monolith C was 189 m^2^ g^−1^, which was higher than those of monoliths A (22 m^2^ g^−1^) and B (74 m^2^ g^−1^), demonstrating that monolith A possessed abundant meso-structure. Figure 3 shows that macro-pores (>1000 nm diameter) existed in all IMSN-C18 hybrid monoliths A, B and C with 15, 25 and 35 mg mL^−1^ of IMSN, respectively. The proportion of smaller pore (1000 *n*m > pore size >50 nm) increased as the concentration of IMSN was increased from 15 to 35 mg mL^−1^, which was also confirmed by the specific surface areas of IMSN-C18 monoliths A (31 m^2^ g^−1^), B (49 m^2^ g^−1^) and C (64 m^2^ g^−1^). Meanwhile, the total intruded volume of monolith C (2.43 cm^3^ g^−1^) was lower than those of monoliths A (2.81 cm^3^ g^−1^) and B (2.9 cm^3^ g^−1^), indicating the smaller pore volume of monolith C. The results from pore structure of the monoliths were consistent with the permeability values (Table 1) and SEM images (Fig. 2 and Supplementary Fig. S1).

### The mechanical stability evaluation of IMSN-C18 monolithic column

The mechanical stabilities of the prepared IMSN-C18 hybrid monolithic columns were examined by connecting the monolithic columns to a nano-UPLC pump (Waters) using water (0.1% TFA) as mobile phase. The permeabilities of monolithic columns were calculated by the Darcy’s law (Equation 1), and listed in Table 1. The equation of Darcy’s law^44^ is as follows:


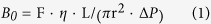


**Figure 4:**
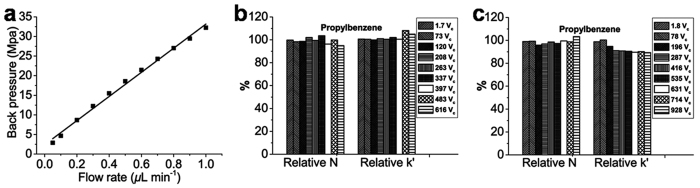
Stability of IMSN-C18 organic-silica hybrid monolithic column. The mechanical stability (**a**) and pH stability (**b**) at pH 1.1, c at pH 11) of IMSN-C18 monolithic column.

Where F is the flow rate of the mobile phase (m^3^ s^−1^), *η* is the dynamic viscosity of the mobile phase (0.001 Pa·s for water at 20 °C), L is the effective length of the column (m), r is the inner radius of the column (m), and ΔP is the pressure drop across the column (Pa). As shown in Fig. 4a, the measured back pressure of monolithic column C increased linearly (R^2^ = 0.994) from 2.9 to 32.2 MPa as the flow rate was increased from 0.05 to 1.0 *μ*L min^−1^, which implied good mechanical stability of the prepared IMSN-C18 capillary hybrid monolithic column. At the same time, the good linear correlations (R^2^ > 0.9999) between the back pressure and the flow rate were also obtained for columns A and B with 15 and 25 mg mL^−1^ of IMSN. However, the calculated ratio of ΔP*/*F for monolithic columns increased with the increase of IMSN concentration, hinting the decline of permeabilities of IMSN-C18 monolithic columns with the increased concentration of IMSN. The calculated permeabilities of columns A, B and C decreased from 4.54 to 1.31 × 10^−14^ m^2^ as the concentration of the IMSN was increased from 15 to 35 mg mL^−1^.

### The pH stability of IMSN-C18 monolithic column

To investigate the pH stability of the IMSN-C18 monolithic column, the column was continuously flushed by 50% ACN with different extreme pH values at the flow rate of 80‒120 *μ*L min^−1^ (before split). The chromatography performance including retention factors (k′ values) and theoretical plate number (N) on the flushed column was monitored by cLC at several intervals. The performance traces of the column after being flushed by 50% ACN at extreme pH values are illustrated in Fig. 4 and Supplementary Fig. S2. Both the retention factors and theoretical plate number remained above 95% when the column was flushed by ca. 600 column volumes of 0.1 mol L^−1^ HCl containing 50% ACN solution (pH 1.1) (Fig. 4b and Supplementary Fig. S2a), indicating the good pH stability of column at low pH value. The IMSN-C18 monolithic column was flushed by 50% ACN at extreme high pH value to test its stability in basic solutions. The retention factors of hydrophobic compounds were about 90% of the original value after flushing the column with ca. 900 column volumes of 10 mmol L^−1^ phosphate solution containing 50% ACN (pH 11) (Fig. 4c and Supplementary Fig. S2b). At this high pH value, theoretical plate number was stable and remained above 94% when the column was flushed by ca. 900 column volumes of the above basic mobile phase (Fig. 4c and Supplementary Fig. S2b). Even 50 mmol L^−1^ phosphate solution containing 50% ACN with a higher pH value of 12 was also applied to test the pH tolerability. The theoretical plate number (N) for the column was higher than 80% of the original value after flushing the column with ca. 260 column volumes of the extreme basic mobile phase (Supplementary Fig. S2c,d). The retention factors (k′ values) decreased to 70% gradually during the flushing period (Supplementary Fig. S2c,d). However, the monolith was flushed out of the capillary with the flushing volume up to ca. 320 column volumes because of the damage of linkage between the monolith and the inner wall of capillary using the strong basic mobile phase. The above results suggested that the IMSN-C18 hybrid monolithic column could be applied under a wide pH range due to the stable structure of the prepared IMSN monolith.

### Reproducibility

The reproducibilities of the IMSN-C18 monolith were assessed through the percent relative standard deviations (RSDs) of retention factors with benzene and toluene as the test compounds on monolithic columns. The run-to-run (n = 4) RSDs were less than 1.2% and 1.1% for benzene and toluene, respectively. The batch to batch (n = 4) RSDs were less than 5.0% and 4.9% for benzene and toluene, respectively. These RSDs indicated that the prepared IMSN-C18 hybrid monolithic column had good stability and reproducibility.

### Chromatographic performance of the IMSN-C18 monolith

**Figure 5:**
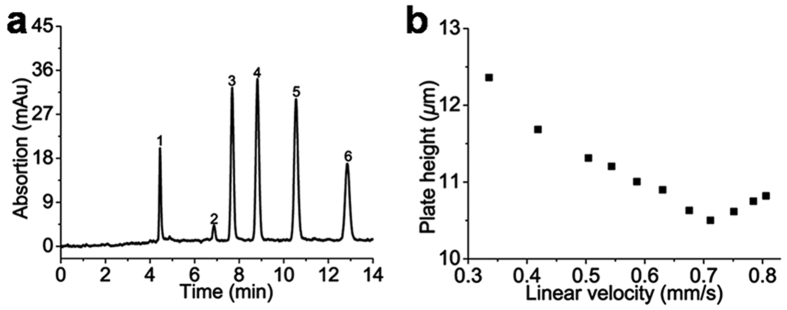
Chromatographic evaluation of IMSN-C18 hybrid monolithic column. (**a**) Separation of alkyl benzenes (1) thiourea, (2) benzene, (3) toluene, (4) ethylbenzene, (5) propylbenzene and (6) butylbenzene on IMSN-C18 hybrid monolithic column C; (**b**) dependence of plate height of butylbenzene on the linear velocity of the mobile phase by cLC on IMSN-C18 hybrid monolithic column C. cLC conditions: column size: (**a,b**) 19 cm × 100 *μ*m i.d.; mobile phase, (**a,b**) ACN/H2O (0.1% FA) (70/30, V/V); flow rate: (**a**) 170 *μ*L min^−1^ (before split); detection wavelength, 214 nm; injection volume: 2 *μ*L sample in split mode.

A test mixture containing thiourea, benzene, toluene, ethylbenzene, propylbenzene and butylbenzene was used to investigate the hydrophobic property of IMSN-C18 hybrid monolithic column. As shown in Fig. 5a, compounds could be separated completely on column C using a mobile phase of ACN/water (0.1% FA) (70/30, v/v), with peaks eluted according to their increased hydrophobicity in the order of thiourea, benzene, toluene, ethylbenzene, propylbenzene, and butylbenzene. A typical RP chromatography retention mechanism was exhibited with these hydrophobic compounds on the IMSN-C18 hybrid monolith. The column efficiency of the IMSN-C18 monolithic column was evaluated by changing the flow rate of the mobile phase. Figure 5b shows the relationship between the flow rate and plate height for butylbenzene. A column efficiency of 95000 plates/m was achieved at the optimal flow rate.

**Figure 6:**
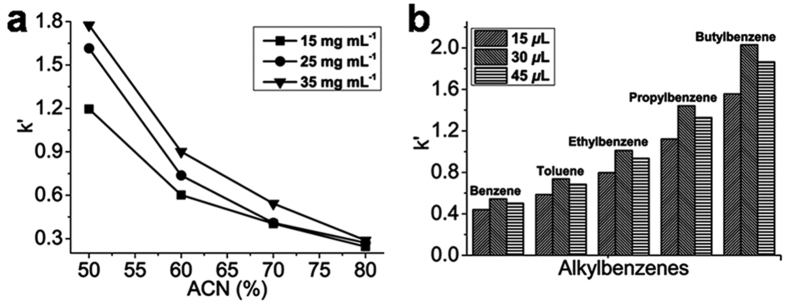
Retention factor on IMSN-C18 hybrid monolithic column. (**a**) Effect of concentration of IMSN on the retention factor of benzene with different content of acetonitrile, (**b**) effect of concentration of C18 monomer on the retention factors of alkylbenzenes under ACN/H2O (0.1% FA) (70/30, V/V) by cLC.

The retention of alkyl benzenes on IMSN-C18 hybrid monolithic columns could be influenced by the structure of monolithic matrix, the amount of the C18 functional groups incorporated onto the silica matrix and ACN content in the mobile phase. As the IMSN crosslinked C18 hybrid monolith was synthesized through a nanoparticle-based “one-pot” approach with IMSN as building block and crosslinker, the effect of IMSN concentration on the retention of alkyl benzenes was carefully investigated. The retention factors of alkyl benzenes on the columns A, B and C were tested. The k′ values of benzene, toluene, ethylbenzene, propylbenzene and butylbenzene generally increased as the concentration of IMSN was increased from 15 to 35 mg mL^−1^(Fig. 6a and Supplementary Fig. S3). This demonstrated that a high concentration of IMSN was conducive to a complete sol-gel reaction between IMSN and VTMS, which resulted in the formation of monolithic columns with a compact silica skeleton and more C18 functional groups. However, it was not appropriate to use excessive IMSN for the preparation of IMSN-C18 monolithic columns since the excessive IMSN was difficult to be pumped into a capillary.

To investigate the effect of C18 monomer content on the retention of alkyl benzenes, IMSN-C18 monolithic columns C, J and K (Table 1) were fabricated with 30, 15 and 45 *μ*L of C18 monomer, respectively. As shown in Fig. 6b and Supplementary Fig. S4, the k′ values of alkyl benzenes increased as the content of C18 monomer was increased from 15 to 30 *μ*L. However, the retention factors of alkyl benzenes were decreased using 45 *μ*L of C18 monomer in the precondensation mixture. These implied that a suitable increase of C18 monomer would be helpful to increase the retention of hydrophobic compounds on the IMSN-C18 monolithic column. The inappropriate amount of C18 monomer could be adverse to the through-pore size and homogeneity of the monolithic column.

The influence of ACN content in the mobile phase on the retention factors (k′ values) of alkyl benzenes was evaluated, with the detailed result displayed in Supplementary section 1 and Supplementary Fig. S5. The IMSN-C18 hybrid monolithic column exhibited a typical hydrophobic-interaction mechanism in separation of alkyl benzenes. In addition, we found that the reverse-phase retention of alkyl benzenes on IMSN-C18 monolithic column C was stronger than that on the reported C18 monoliths formed by ordinary and non-hydrolytic sol-gel reaction through “one-pot” method^29^,^45^. This could be attributed to more incorporated vinyl functional groups for the nanoparticle-based sol-gel method than those for ordinary and non-hydrolytic sol-gel approach.

### Separations on IMSN-C18 hybrid monolithic column

**Figure 7:**
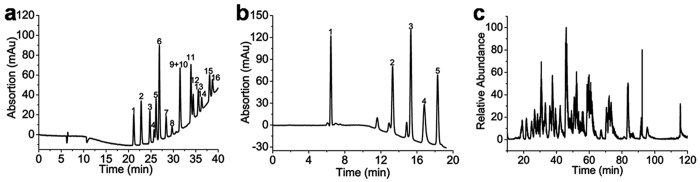
Separations on IMSN-C18 hybrid monolithic column. (**a**) Separation of EPA 610 on IMSN-C18 hybrid monolithic column C by cLC; Analytes: (**a**) (1) naphthalene, (2) acenaphthylene, (3) acenaphthene, (4) fluorene, (5) phenanthrene, (6) anthracene, (7) fluoranthene, (8) pyrene, (9) benzo (**a**) anthracene, (10) chrysene, (11) benzo (**b**) fluoranthene, (12) benzo (**k**) fluoranthene, (13) benzo (**a**) pyrene, (14) dibenzo (**a,h**) anthracene, (15) benzo (**g,h,i**) perylene and (16) indeno(1,2,3-cd)pyrene; (**b**) separation of a mixture of proteins (1) Ribonuclease B, (2) Cytochrome C, (3) Lysozyme, (4) Albumin from bovine serum and (5) Myoglobin on IMSN-C18 hybrid monolithic column C; (**c**) base-peak chromatogram of cLC-MS/MS analysis of a BSA tryptic digest on IMSN-C18 monolithic column C. Separation conditions: column size: (**a,b**) 19 cm × 100 *μ*m i.d. and (**c**) 24 cm × 100 *μ*m i.d.; detection wavelength, (**a**) 254 and (**b**) 214 nm; mobile phase A: water containing 0.1% FA for (a) and 0.1% TFA for (**b,**c), mobile phase B: ACN containing 0.1% FA for a and 0.1% TFA for (**b,c**); gradient elution, (**a**) 50% to 100% B in 30 min, (**b**): 0‒1 min 40% B, 1‒21 min 40% to 60% B, 21‒30 min 60% B, (**c**) 5% to 35% B over 90 min; flow rate: (**a,b**) 0.12 and (**c**) 0.08 mL min^−1^ before split; injection volume: (**a,b**) 2 and (**c**) 20 *μ*L in split mode.

The IMSN-C18 hybrid monolithic column was first applied for the separation of alkyl benzenes by cLC. The representative separation chromatogram is shown in Fig. 5a. The effects of IMSN concentration and the amount of C18 monomer on the retention factors of alkyl benzenes are shown in Fig. 6a,b and Supplementary Figs S3 and S4. EPA 610 that consists of 16 priority pollutant PAHs and presents potential health hazards was used to evaluate the separation ability of the IMSN-C18 hybrid monolithic column by gradient elution. Figure 7a shows that a good separation of EPA 610 could be achieved except benzo(*a*)anthracene (analyte 9) and chrysene (analyte 10), confirming the reverse-phase retention behavior and the good separation ability of the IMSN-C18 monolithic cloumn. To further validate the separation potential of the IMSN-C18 monolithic column, a mixture of standard proteins was selected as the analytes for cLC. The chromatogram shown in Fig. 7b indicates that the IMSN-C18 monolithic column is capable of separating big biomolecules and possesses the potentials for the “top-down” proteomics.

### Tryptic digestions analysis on IMSN-C18 hybrid monolithic column

The hybrid monolithic column was also evaluated by cLC-MS/MS separation of BSA tryptic digests. The cLC-MS/MS analysis was performed under a RP mode with 20 *μ*L of BSA digest loaded on a IMSN-C18 hybrid monolithic column (24 cm × 100 *μ*m i.d.). As a comparison, a homemade C18-particle-packed capillary column (14 cm × 75 *μ*m i.d) was also applied to separate BSA tryptic digests under the same conditions. The base peak chromatogram is illustrated in Fig. 7c and Supplementary Fig. S6. On the basis of database search of the chromatogram of BSA tryptic digests, the identified number of unique peptides and the protein sequence coverage by the IMSN-C18 hybrid monolithic column were 68 (RSD = 2.9%, n = 3) and 87.5% (RSD = 1.4%, n = 3), respectively, which were almost the same as those by a C18-particle-packed column with 72 (RSD = 3.9%, n = 3) unique peptides and 83% (RSD = 2.4%, n = 3) protein coverage. Therefore, the IMSN-C18 monolithic column showed great potentials in the separation of tryptic digests of proteins for “bottom-up” proteomic application.

## Discussion

IMSN crosslinked C18 capillary hybrid monolithic columns have been fabricated via “one-pot” approach in which the intact mesoporous silica nanoparticle was used as the crosslinker and building block. The morphologies of IMSN-C18 monolithic column were affected by the fabrication conditions, such as the concentration of IMSN, the polycondensation temperature, the amount of PEG and the contents of VTMS and C18 monomer. SEM images imply that a more uniform monolithic structure could be formed using a higher concentration of IMSN. In addition, the N_2_ adsorption/desorption and mercury intrusion porosimetry experiments revealed that the IMSN-C18 monolith prepared by 35 mg mL^−1^ of IMSN possessed the highest specific surface area and the most abundant micro/meso-structure. This could be attributed to a more complete sol-gel reaction between IMSN and VTMS with a higher concentration of IMSN. Moreover, the chromatography performance (theoretical plate number and retention factors) for alkylbenzenes on the IMSN-C18 monolithic column also verified the consequences from the pore structure and SEM images. Thus, a higher IMSN concentration was conducive for the formation of IMSN-C18 monolithic column with a uniform morphology, abundant micro/meso-structure and good chromatography performance. However, the excessive IMSN was difficult to be pumped into capillary and would result in the decrease of permeability of the IMSN-C18 monolithic column. Therefore, it is particularly important to adjust the concentration of IMSN to prepare the IMSN-C18 hybrid monolithic column. The nonuniform morphologies and/or the inferior column efficiencies would be obtained with inappropriate amount of PEG, VTMS and C18 monomer, as well as improper fabrication temperature ascribe to the inhomogeneous sol-gel reaction. Thus, careful adjustments of these preparation conditions are necessary to prepare a uniform IMSN-C18 monolithic column with good column efficiency. The IMSN crosslinked C18 monolithic column exhibited good mechanical stability and pH stability in a wide pH range, which could be owing to the stable structure of the monolithic skeleton formed by the nanoparticle-based “one-pot” approach. The abundant micro/meso-structures of the IMSN-C18 monolithic column was suitable for separation of small molecules and macrobiomolecules. The successful preparation and good chromatographic performance of the resulting IMSN-C18 monolith represent a more extensive and promising application of the nanoparticle-based “one-pot” approach, with IMSN as building block and crosslinker.

## Methods

### Chemicals and materials

All regents were analytical grade unless otherwise stated and are listed in detail in Supplementary section 2.

### Preparation of intact mesoporous silica nanoparticle

Intact mesoporous silica nanoparticles (IMSNs) were synthesized according to the method by Meng^46^. The detailed experiment conditions are provided in Supplementary section 3. As shown in Supplementary Fig. S7, monodisperse intact mesoporous silica nanoparticles with diameter of 100‒130 nm were successfully synthesized.

### Preparation of IMSN-C18 organic-silica hybrid monolithic column

To prepare the IMSN-C18 hybrid monolithic column, IMSNs were dissolved in porogenic solvent, which consisted of n-propanol (220 *μ*L), PEG (5 mg) and 0.01 mol L^−1^ acetic acid solution (26 *μ*L), to create homogeneous dispersion under ultrasonication for 20 min. VTMS (104 *μ*L) was added to the resultant mixture with 3 min sonification, followed by the addition of azosiobutyronitrile (1 mg) and C18 monomer (30 *μ*L). After further ultrasonication for 10 min, the homogeneous mixture was introduced into a fused-silica capillary with an appropriate length by nitrogen pressure. Both ends of the filled capillary were sealed with two pieces of rubber, and the capillary was incubated at 40 °C for 12 h and then 60 °C for 12 h, for the condensation and polymerization, respectively. The obtained IMSN-C18 monolithic column was flushed with methanol to remove the PEG and other residuals. In addition, the monoliths were also prepared in tube and washed by ethanol.

### Capillary liquid chromatography (cLC)

The chromatographic investigations on the IMSN-C18 hybrid monolithic columns were performed on a high performance liquid chromatography (HPLC) system including two LC-10AD VP pumps (shimadzu, Kyoto, Japan) and a UV detector (K-2501, Beijing Huayanglimin instrument Co., Ltd, Beijing, China). Data were collected at 214/254 nm and processed by chromatography workstation (HW-2000, Shanghai Qianpu software Co., Ltd, Shanghai, China). A Rheodyne 8125 (Index Health & science LLC, Rohnert Park, USA) with a 5 *μ*L sample loop and A DG230-2 online degasser (Dalian Elite Analytical Instruments, Co., Ltd, Dalian China) were used. A T-union connector served as splitter with one end connected to a capillary monolithic column and another end to a blank capillary (50 *μ*m i.d. and 365 *μ*m o.d.) with appropriate length. The split ratio was controlled at about 100‒800:1. The outlet of the capillary monolithic column was collected by a Teflon tube to an empty fused-silica capillary (50 *μ*m i.d. and 365 *μ*m o.d.), where a detection window was made by removing a 2 mm length of polyamide in a position of ca. 5 cm from the separation monolithic column outlet. The retention factor (k′ values) was defined as equation (2):


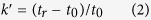


Where t_r_ and t_0_ represent the retention time of analyte and unretained compound, respectively.

### Characterization of IMSN-C18 monolithic column

The detailed characterization experiments of the IMSN-C18 monolithic column is in Supplementary section 4.

### Tryptic digest of BSA and cLC-MS/MS analysis

The tryptic digest of BSA and cLC-MS/MS analysis were performed according to the procedure previously reported with the minor modification^38^. Detailed experimental procedures are provided in the Supplementary section 5.

## Additional Information

**How to cite this article**: Liu, S. *et al.* One-Pot Approach to Prepare Organo-silica Hybrid Capillary Monolithic Column with Intact Mesoporous Silica Nanoparticle as Building Block. *Sci. Rep.* **6**, 34718; doi: 10.1038/srep34718 (2016).

## Supplementary Material

Supplementary Information

## Figures and Tables

**Table 1:**
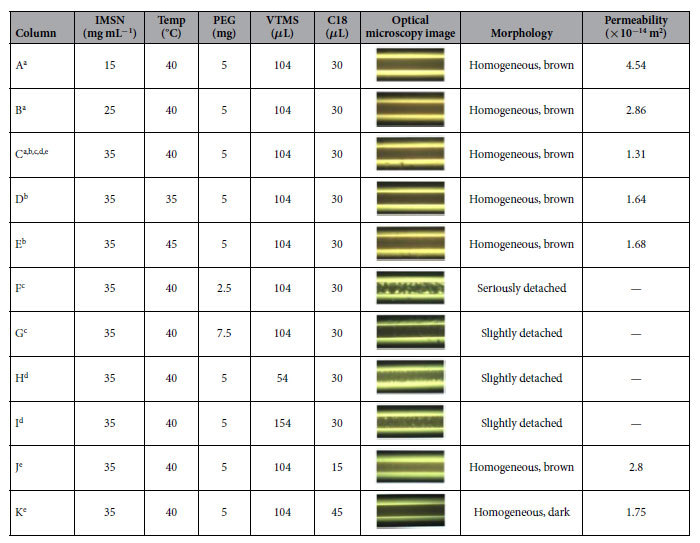
Parameters (a: IMSN concentration, b: temperature, c: PEG content, d: VTMS content and e: C18 content) for preparation of IMSN-C18 organic-inorganic hybrid monolithic column.
