# Supplementary material for: One-Pot Approach to Prepare Organo-silica Hybrid Capillary Monolithic Column with Intact Mesoporous Silica Nanoparticle as Building Block
Source: Sci Rep. 2016 Oct 4;6:34718. doi: 10.1038/srep34718 (PMC5048148; doi:10.1038/srep34718)
Supplement: Supplementary Information [file srep34718-s1.pdf]

# Supplementary Materials

## One-Pot Approach to Prepare Organo-silica Hybrid Capillary Monolithic Column with Intact Mesoporous Silica Nanoparticle as Building Block

**Shengju Liu[1,2], Jiaxi Peng[1,2], Zheyi Liu[1,2], Zhongshan Liu[1,2], Hongyan Zhang[1,2], Ren'an Wu[1,*]**

[1]CAS Key Laboratory of Separation Science for Analytical Chemistry, National Chromatographic R & A Center, Dalian Institute of Chemical Physics, Chinese Academy of Sciences (CAS), Dalian 116023, China.

[2]University of Chinese Academy of Sciences, Beijing 100049, China.

*Corresponding author: wurenan@dicp.ac.cn (Dr. Ren’an Wu)

**Supplementary section 1. The effect of ACN content on the retention of alkyl benzenes on IMSN-C18 hybrid monolithic column**. The influence of ACN content in the mobile phase on the retention factors (k' values) of alkyl benzenes was evaluated. As displayed in Figure S5, the results indicated that the retention factors of benzene, toluene, ethylbenzene, propylbenzene and butylbenzene decreased as the ACN content was increased from 50% to 80%. Butylbenzene showed the strongest retention on the non-polar IMSN-C18 monolithic column C due to its highest hydrophobicity among the five test compounds. The column exhibited good retention and separation ability for neutral compounds as these five compounds were baseline-separated even in a mobile phase of 80% (v/v) ACN/water. These results indicated that the IMSN-C18 monolithic column possessed a typical hydrophobic-interaction mechanism for separation of these five compounds.

**Supplementary section 2. Chemicals and materials:** All regents were analytical grade unless otherwise stated. Tetraethyl orthosilicate (TEOS), vinyltrimethoxysilane (VTMS), poly(ethylene glycol) (PEG, Mw=10000), stearyl methacrylate (C18 monomer) and EPA 610 were purchased from Aldrich. Hexadecyltrimethylammonium bromide (CTAB), bovine serum albumin (BSA), lysozyme, cytochrome C, myoglobin, ribonuclease B, trypsin, dithiothreitol (DTT), (TFA) and iodoacetamide (IAA) were purchased from Sigma. Azosiobutyronitrile (AIBN) was obtained from J&K Scientific Ltd. (Beijing, China). Acetic acid (HPLC grade) was obtained from Tianjin Kermal chemical reagent Co. Ltd. (Tianjin, China). C18 particles (5 $\mu$m, 120 Å pore) was purchased from SunChrom (USA). Fused-silica capillaries with dimensions of 100 $\mu$m i.d. and 365 $\mu$m o.d. were obtained from Refine Chromatography Ltd. (Yongnian, Hebei, China). HPLC-grade ACN was used for the preparation of mobile phases. The water used in all

experiments was doubly distilled and purified by Milli-Q system (Millipore Inc., Milford, MA) unless otherwise stated. Other reagents were obtained as received.

**Supplementary section 3. Preparation of intact mesoporous silica nanoparticle.** Intact mesoporous silica nanoparticle (IMSN) was synthesized according to the method by Meng.[1] 250 mg of CTAB were dissolved in 120 mL of deionized water, followed by the addition of 875 $\mu$L of 2 mol $L^{-1}$ NaOH aqueous solution. The CTAB solution was heated to and kept at 80 ℃ for half an hour before 1.25 mL of TEOS was added. The reaction was kept at 80 ℃ for another 2 hours. The resulting nanoparticles were dried at 60 ℃ under vacuum overnight after centrifuging and washing four times with deionized water and ethanol, respectively. As seen from Fig. S7, monodisperse intact mesoporous silica nanoparticles with diameter of 100–130 nm were successfully synthesized.

**Supplementary section 4. Characterization of IMSN-C18 monolithic column.** Scanning electron microscopy (SEM) images were obtained using JEOL JSM-5600 scanning electron microscopy (JEOL, Tokyo, Japan). Transmission electron microscopy (TEM) images were obtained on JEM-2000 EX microscope (JEOL, Japan) operated at 120 KV. The pore structure datum of IMSN-C18 monolith were obtained through Nitrogen adsorption/desorption and Mercury intrusion measurements. Prior to test, bulk materials (2.0 g) were also polymerized under the same conditions as the preparation of the monolithic capillary columns. To remove the residuals, the synthesized monoliths were dipped into 120 mL of ethanol and shaken for 12 h at room temperature. The cleaning step was repeated for 6 times. Ultimately, the synthesized monoliths were dried under vacuum at 60 ℃ for 24 h. The specific surface areas obtained from

Nitrogen adsorption/desorption data was attained on a Quadrasorb SI surface area analyzer (Quantachrome, Boynton Beach, USA). The samples were dried at 100 ℃ for 10 h before analysis. The total surface areas were calculated by Brunauer-Emmett-Teller (BET) model. The micro-pore and meso-pore surface areas were derived from the t-plot. Mercury intrusion porosimetry was accomplished on Pore Master GT-60 (America Quantachrome Instrument Corporation, USA). Prior to the experiments, the samples were dried at 110 ℃ for 8 h. Pore diameters were calculated according to the Washburn equation:

$$pd = -4\gamma \cdot \cos\theta \text{ , Equation (1)}$$

Where $p$ is the applied pressure, $d$ is the radius of a pore opening, $\gamma$ is the surface tension of mercury (0.4842 N/m at 25°C) and $\theta$ is the contact angle between mercury and the sample. The contact angle ranges from 135° to 142°.

**Supplementary section 5. The tryptic digest of BSA and cLC-MS/MS analysis.** BSA (10 mg) was dissolved in 5 mL of denaturing buffer containing urea (8 mol $L^{-1}$) and ammonium bicarbonate (100 mmol $L^{-1}$). After the addition of 100 $\mu$L of DTT (1 mol $L^{-1}$), the mixture was incubated at 60 ℃ for 1 h to reduce the disulfide bonds of the protein, and then cooled to room temperature; subsequently, 200 $\mu$L of IAA (1 mol $L^{-1}$) was added, and the mixture was then incubated at room temperature in dark for 40 min; Finally, the mixture was diluted by 8-fold with 100 mmol $L^{-1}$ ammonium bicarbonate buffer (pH 8.2) and digested at 37 ℃ for 17 h by trypsin with the ratio of enzyme-to-substrate of 1:25 (w/w). The pH value of the obtained tryptic digestion solution was adjusted to ca. 2.7 with 10% trifluoroacetic acid (TFA) aqueous solution. Successively, the BSA tryptic digestion solution was solid-phase extracted with a homemade C18

cartridge to remove salts which were introduced in the above process. At last, the BSA digests were dried in vacuum concentrator and dispersed in 0.1% formic acid (FA) with the concentration of 0.05 mg $mL^{-1}$ before cLC-MS/MS analysis. The tryptic digest of BSA and cLC-MS/MS analysis were performed according to the procedure previously reported with the minor modification.[2] The Tryptic digest was trapped on a homemade C18-particle-packed trap column (3 cm length × 200 $\mu$m i.d.), and the subsequent separation was carried out on the IMSN-C18 hybrid capillary monolithic column (24 cm length × 100 $\mu$m i.d.) or C18-particle-packed capillary column (14 cm × 75 $\mu$m i.d.) with an integrated emitter, which was prepared by directly tapering the tip from the outlet of the capillary.

The cLC-MS/MS experiments were performed by interfacing a surveyor MS pump to a Finnigan LTQ ion trap mass spectrometer (Finnigan MAT, Thermofinnigan, San Jose, CA). Mobile phase A was water (containing 0.1% formic acid), and mobile phase B was ACN (containing 0.1% formic acid). A homemade C18-particle-packed capillary column (3 cm × 200 $\mu$m i.d.) was used as a trap column for sample injection and connected to analytical column with a T-union connector. The BSA tryptic digests were automatically injected onto the column with mobile phase A for 20 min. After then, the trapped peptides were separated on the IMSN-C18 hybrid monolithic column (24 cm×100 $\mu$m i.d.) or C18 particle-packed capillary column (14 cm × 75 $\mu$m i.d.) with an integrated emitter, which was prepared by directly tapering the tip from the outlet of the monolithic capillary column[3]. The reversal separation was performed on the IMSN-C18 hybrid monolithic column with gradient elution from 5 to 35% ACN (containing 0.1% formic acid) within 90 min. The LTQ linear ion trap mass spectrometer equipped with a nanospray ion source. The temperature of the ion transfer capillary was set at 200 ℃. The spray

voltage was set at 1.8 kV, and the normalized collision energy was set at 35.0%. One microscan was set for each MS and MS/MS scan. All MS and MS/MS spectra were acquired in the data dependent mode. The mass spectrometer was set that one full MS scan was followed by six MS/MS scans on the six most intense ions. The dynamic exclusion function was set as follows: repeat count 2, repeat duration 30 s, and exclusion duration 90 s. System control and data collection were done by Xcalibur software version 1.4 (Thermo). The scan range was set from m/z 400 to m/z 1600.

The acquired MS/MS spectra were searched on a database using the MASCOT (version 2.2.04) protein identification platform (Matrix Science, London, UK), and the MS/MS spectra of pull-down were searched against IPI bovine BOVIN 3.32 (32,946 sequences; 16,109,453 residues). Cysteine residues were searched as fixed modification of 57.0215 Da, and methionine residues as variable modification of 15.9949 Da. Peptides were searched using fully tryptic cleavage constraints and up to two internal cleavages sites were allowed for tryptic digestion. The mass tolerances were 2 Da for parent masses and 1 Da for fragment masses.

Supplementary Figure St. SEM images of IMSN-C18 organic-silica hybrid monolithic column (a) ColumnA, (b) Column B, (c) Column D, (d) Column E, (e) Column J, (f) Column K.

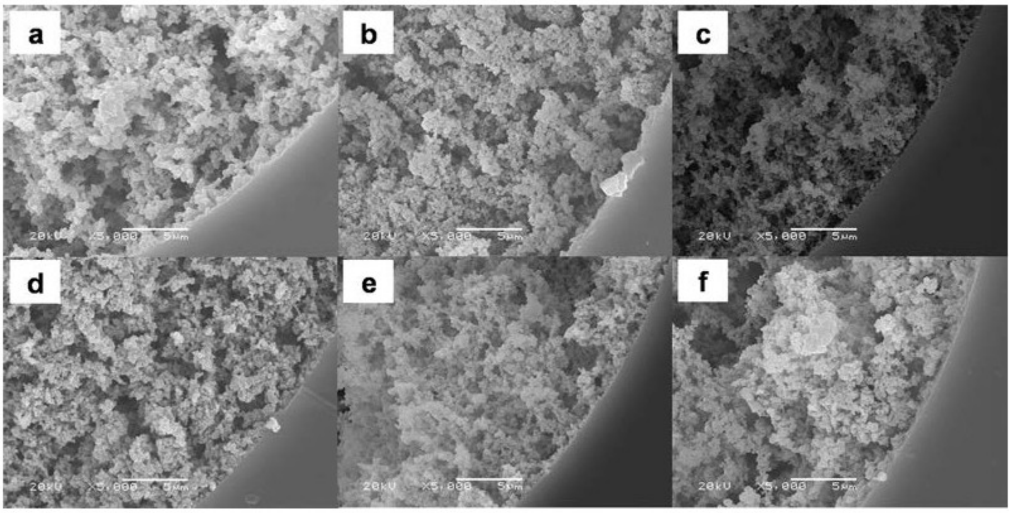

**Supplementary Figure S2.** The pH stability of IMSN-C18 hybrid monolithic column: (a) flushed by 100 mmol $L^{-1}$ of HCl solution containing 50% ACN at pH 1.1; (b) flushed by 10 mmol $L^{-1}$ of phosphate solution containing 50% ACN at pH 11; (c, d) flushed by 50 mmol $L^{-1}$ of phosphate solution containing 50% ACN at pH 12, flushing flow rate 80–120 $\mu$L $min^{-1}$ (before split); cLC conditions: mobile phase, the same as the above flushing mobile phase, respectively; flow rate: 120 $\mu$L $min^{-1}$ (before split); detection wavelength, 214 nm.

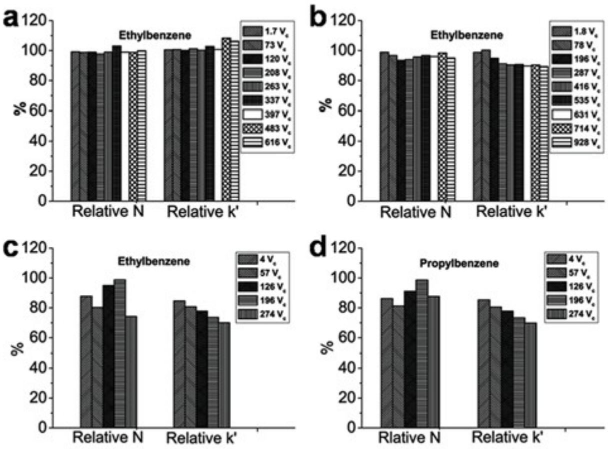

**Supplementary Figure S3.** Effect of concentration of intact mesoporous nanoparticle on the retention factors of (a) toluene, (b) ethylbenzene, (c) propylbenzene and (d) butylbenzene with different content of acetonitrile by cLC on IMSN-C18 hybrid monolithic column, separation conditions: mobile phase A: water (0.1% FA), mobile phase B: ACN; detection wavelength: 214 nm; inject 2 $\mu$L samples in split mode.

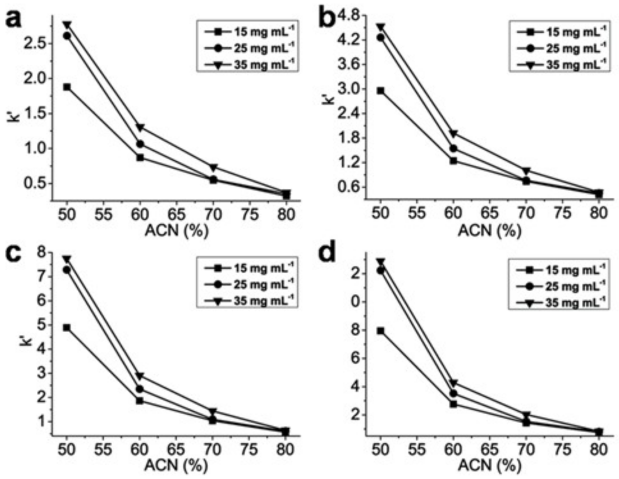


**Supplementary Figure S4.** Effect of concentration of C18 monomer on the retention factors of alkyl benzenes under ACN/H2O (0.1% FA) (60/40, V/V) by cLC on IMSN-C18 hybrid monolithic column.

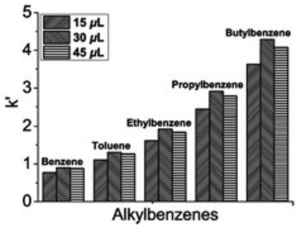

**Supplementary Figure S5.** The effect of ACN content in the mobile phase on the retention factors of alkyl benzenes on IMSN-C18 hybrid monolithic column C by cLC; separation conditions: column size: 19 cm × 100 $\mu$m i.d.; mobile phase A: water (0.1% FA); mobile phase B: ACN; detection wavelength: 214 nm; inject 2 $\mu$L samples in split mode and flow rate is 140 $\mu$L min$^{-1}$ (before split).

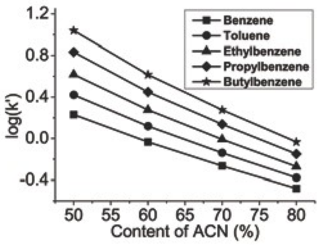


**Supplementary Figure S6.** Base-peak chromatogram of cLC-MS/MS analysis of a BSA tryptic digest on C18-particle-packed column; experiment conditions: column size, 14 cm × 75 $\mu$m i.d.; mobile phase A: 100% water (0.1% FA), B: 100% ACN (0.1% FA); gradient: 5% to 35% B over 90 min; flow rate: 80 $\mu$L min$^{-1}$ (before split).

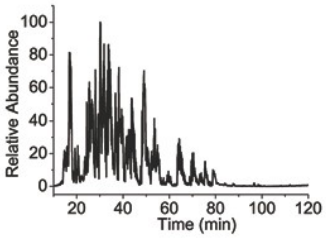

**Supplementary Figure S7.** TEM images of intact mesoporous silica nanoparticles.

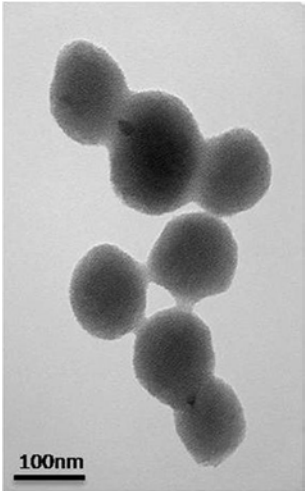


**Supplementary Table S1.** The BET surface areas of the IMSN-C18 hybrid monoliths A, B, C obtained from $N_2$ adsorption and desorption experiment.

| Monolith | A | B | C |
|---|---|---|---|
| BET surface area ($m^2$ $g^{-1}$) | 22 | 74 | 188 |

**Supplementary Table S2.** The specific surface areas and total intruded volumes of the IMSN-C18 hybrid monoliths A, B, C by the mercury intrusion method.

| Monolith | A | B | C |
|---|---|---|---|
| Specific surface area ($m^2$ $g^{-1}$) | 31 | 49 | 64 |
| Total intruded volume ($cm^3$ $g^{-1}$) | 2.81 | 2.9 | 2.43 |

# Reference


1. Meng, H. *et al.* Use of size and a copolymer design feature to improve the biodistribution and the enhanced permeability and retention effect of doxorubicin-loaded mesoporous silica nanoparticles in a murine xenograft tumor model. *ACS Nano* **5**, 4131-4144 (2011).
2. Wu, M. H. *et al.* Polyhedral oligomeric silsesquioxane as a cross-linker for preparation of inorganic-organic hybrid monolithic columns. *Ana. Chem.* **82**, 5447-5454 (2010).
3. Xie, C. H., Ye, M. L., Jiang, X. G., Jin, W. H. & Zou, H. F. Octadecylated silica monolith capillary column with integrated nanoelectrospray ionization emitter for highly efficient proteome analysis. *Mol. Cell. Prote.* **5**, 454-461 (2006).
